# Assessment of agro-morphological variability of dry-season sorghum cultivars in Chad as novel sources of drought tolerance

**DOI:** 10.1038/s41598-019-56192-6

**Published:** 2019-12-20

**Authors:** Gapili Naoura, Nerbewende Sawadogo, Eyanawa A. Atchozou, Yves Emendack, Mahamat A. Hassan, Djinodji Reoungal, Doyam N. Amos, Nadjiam Djirabaye, Ramadjita Tabo, Haydee Laza

**Affiliations:** 1 Institut Tchadien de Recherche Agronomique pour le Développement (ITRAD), B.P. 5400 N’Djaména, Tchad; 2 Equipe Génétique et Amélioration des plantes, Laboratoire Biosciences, Université Ouaga I Pr Joseph KI-ZERBO 03 BP 7021, Ouagadougou, 03 Burkina Faso; 3 Institut Togolais de Recherche Agronomique, Lome, Togo; 4 USDA-ARS, Cropping Systems Research Laboratory, Lubbock, TX USA; 5 International Crops Research Institute for the Semi-Arid Tropics (ICRISAT), Bamako, BP 320 Bamako, Mali

**Keywords:** Plant breeding, Plant stress responses, Plant physiology

## Abstract

Dry-season sorghum is a type of sorghum whose establishment ends at the end of the rainy season and its development takes place during the dry and cold harmattan period. Its root system is particularly well developed with deep penetration for water withdrawal. This study was conducted to assess the level of genetic diversity present among dry-season sorghum in Chad’s Sudanese zone using phenotypic traits, and to identify new sources of drought tolerance that could be used in sorghum breeding programs. A high variability in qualitative traits was observed except for the botanical race which showed that all cultivars were of *durra* race. It was also observed that most cultivars had compact panicles (66.67%), mostly black glumes (66.67%), glume hairiness (58.33%) and did not have aristation (91.67%). Most qualitative traits showed a coefficient of variation of less than 30%, and the analysis of the variance showed that at 0.1% probability, there were significant differences between cultivars for all traits except botanical race. It was observed that the potential productivity of dry-season sorghum of this collection was strongly related to their staygreen characteristic; a trait of enormous importance in breeding for postflowering drought tolerance in sorghum. Plant height was highly heritable (91.9%), followed by the peduncle length (90.2%), panicle length (87.5%) and the internodes number (86.5%). Structuring of diversity separated the cultivars into four statistically distinct groups; with group 2 clustering cultivars with panicle productivity, early maturity and high staygreen, and other traits that contribute to the performance of cultivars. The findings will help to enhance the selection and production of dry-season sorghum in Chad and also provide alternative sources for staygreen introgression into the larger sorghum breeding community.

## Introduction

Sorghum [*Sorghum bicolor* (L.) Moench] is a major staple food and fodder crop in tropical and semi-tropical Africa and Asia^1^. It is the fifth most important cereal crop worldwide after wheat, rice, maize and barley; and is widely grown in the subtropical and tropical zones of the continent of Africa. Its annual global production is between 57 and 63 million tons^2^, with 23,350,064tons produced on about 23,142,595 ha in Africa^3^. In tropical regions, sorghum is grown largely as a rainfed crop during the fall season or on residual soil moisture after river floods^4^. Dry-season sorghum; also know as post-rainy season sorghum, is a type of sorghum which grows on residual soil moisture^5–10^.

In Africa dry-season sorghum accounts for about 2 million hectares of the total sorghum production hectarage^11^. It is mostly cultivated in west and central Africa particularly in three main areas: the Senegal river basin, the Niger and Mali delta and the Lake Chad basin^7^. In Chad dry-season sorghum is the third most important cereal crop, both in terms of area planted and production. Over the last ten years, the average annual production has been 112,800 tons on an average area of 130,000 hectares contributing 12% of total cereal production with less than 9% of the total cereal area^12^. According to^13^, in Chad, dry-season sorghum (locally know as Berbere) is considered an important cereal crop with good grain quality.

Dry-season sorghum is particularly cultivated at the ending of the rain season, on land that is still drained of water fromprolonged submersion in the rainy season^14^, or following a withdrawal of floods from surface streams. Often referred to as transplanted sorghum (i.e. grown by transplanting), dry-season sorghum are transplanted on vertisol at the end of the rainy season or on hydromorphic soil before the end of the rainy season^6^. According to^15^, this cereal crop grows very well on Vertisol (30–60% clay), heavy and light alluviums, red, gray, yellow loams and sandy soil. Thus, the plant grows without rain, drawing water from deeper soil profiles thanks to its deep root system. Since the cultivation of the dry-season sorghum is carried out after the withdrawal of flood waters, the intensity and the characteristics of the flood determines the yearly yield potential.

When cereal crops are subjected to an increasing environmental or biotic stress after anthesis (terminal stress), the most prominent result is the poor grain filling and the consequent loss in yield^16^. According to^17^ drought stress is a serious agronomic problem contributing to severe yield losses worldwide. This is not the case for the dry- season sorghum The staygreen trait gives plants the ability to retain greenness during grain ripening under water limited conditions^18,19^ and this trait has been associated to drought tolerance^20,21^. Due to their ability to grown only under residual moisture soil, dry-season sorghum has a high resistance to drought^22^. Thus, dry-season sorghum with good drought tolerance could be a good source of genes to breed for drought tolerance and increase productivity of this and other types of sorghum in Chad and in the global sorghum breeding community at large.

Prospecting is one of the ways, often the only way, to save endangered species^23^. To relieve losses in species diversity, a dryseason sorghum survey of the Chadian Sudanese zone was carried out and resulted in the collection of cultivars preserved in the ITRAD germplasm^24^. The assessment of germplasm is essential for using the diversity available in the breeding program of any crop^25^. Despite the importance of dry-season sorghum in Chad, very little research has been done on genetic diversity of the germplasm. This study was carried out to evaluate the genetic variability available in this collection and allow the identification of high-performance cultivars and identify traits of interest for a breeding program.

## Materials and Methods

### Plant material

Fifty six cultivars of dry-season sorghum from the Bébédjia germplasm were used for this study (Table 1). The genotypes in this germplasm were collected in 2012 at three important zones of production of the dry-season sorghum in southern Chad (18 in Moyo Kebbi East; MKE, 15 in Mayo Kebbi West; MKW and 22 in Tandjilé; TDJ).

**Table 1:** Dry-season sorghum cultivar names and annual rainfall of zones.

Name	Regions	Villages	Rainfall 2008–2018 (mm)	Rainfall 2018 (mm)
“Maïdekomi”	TDJ	Doumougou	*1,080.8*	*1,018*
“Farine Kouamgue”	TDJ	Doumougou	*1,080.8*	*1,018*
“Wakba”	TDJ	Doumougou	*1,080.8*	*1,018*
“Farine”	TDJ	Doumougou	*1,080.8*	*1,018*
“Beurdague”	TDJ	Doumougou	*1,080.8*	*1,018*
“Bouagué”	TDJ	Doumougou	*1,080.8*	*1,018*
“Badourou”	TDJ	Doumougou	*1,080.8*	*1,018*
“Toumon guessiri”	TDJ	Doumougou	*1,080.8*	*1,018*
“farine gournagourna”	TDJ	Tchaguine	*1,080.8*	*1,018*
“Gogoum jaune”	TDJ	Tchaguine	*1,080.8*	*1,018*
“Bagouwaye élété”	TDJ	Tchaguine	*1,080.8*	*1,018*
“Toukom -Toumon”	TDJ	Tchaguine	*1,080.8*	*1,018*
“Secheresse”	TDJ	Tchaguine	*1,080.8*	*1,018*
“Toumon Toukom”	TDJ	Tchaguine	*1,080.8*	*1,018*
“Dormi rouge”	TDJ	Tchaguine	*1,080.8*	*1,018*
“farine gogmi rouge”	TDJ	Tchaguine	*1,080.8*	*1,018*
“Farine blanc”	TDJ	Tchaguine	*1,080.8*	*1,018*
“Secheresse blanc”	TDJ	Tchaguine	*1,080.8*	*1,018*
“Gogoumi farine blanc”	TDJ	Tchaguine	*1,080.8*	*1,018*
“Bagouwaye”	TDJ	Tchaguine	*1,080.8*	*1,018*
“Heda dallatcho”	TDJ	Tchaguine	*1,080.8*	*1,018*
“Dormi”	TDJ	Tchaguine	*1,080.8*	*1,018*
“Dinglenga”	MKE	Goulmourévé	966.7	930.1
“Tchokowawana”	MKE	Goulmourévé	966.7	930.1
“Safrana Madawaïna”	MKE	Goulmourévé	966.7	930.1
“Bourbourina”	MKE	Goulmourévé	966.7	930.1
“Bourbourina 2”	MKE	Goulmourévé	966.7	930.1
“Medjeriana”	MKE	Goulmourévé	966.7	930.1
“Ndjamena 2”	MKE	Goulmourévé	966.7	930.1
“Safradjou gomna”	MKE	Goulmourévé	966.7	930.1
“Djogomna”	MKE	Goulmourévé	966.7	930.1
“Ndjamena”	MKE	Goulmourévé	966.7	930.1
“Babou rouge”	MKE	Fianga	892.3	1,028.2
“Mindeurie”	MKE	Fianga	892.3	1,028.2
“Babou blanc”	MKE	Fianga	892.3	1,028.2
“Glinding”	MKE	Fianga	892.3	1,028.2
“Tchorolalé”	MKE	Fianga	892.3	1,028.2
“Donglon”	MKE	Fianga	892.3	1,028.2
“Donglon rouge”	MKE	Fianga	892.3	1,028.2
“Manbalam”	MKE	Fianga	892.3	1,028.2
“Vounging a 2 grains”	MKW	Margalao	*1,118*	*1,044.2*
“var 42”	MKW	Margalao	*1,118*	*1,044.2*
“Donglo Bando”	MKW	Margalao	*1,118*	*1,044.2*
“Donglo Bando 2”	MKW	Margalao	*1,118*	*1,044.2*
“Vounging Membou”	MKW	Margalao	*1,118*	*1,044.2*
“Bambou mbou”	MKW	Margalao	*1,118*	*1,044.2*
“Bambou”	MKW	Margalao	*1,118*	*1,044.2*
“Sorghum”	MKW	Margalao	*1,118*	*1,044.2*
“Zabili”	MKW	Margalao	*1,118*	*1,044.2*
“Sakadassaré”	MKW	Biparé	*1,010.52*	*957.2*
“Dagadourou”	MKW	Biparé	*1,010.52*	*957.2*
“Dalassi”	MKW	Biparé	*1,010.52*	*957.2*
“Gbor”	MKW	Biparé	*1,010.52*	*957.2*
“Maï Mamou”	MKW	Biparé	*1,010.52*	*957.2*
“Madjéri”	MKW	Biparé	*1,010.52*	*957.2*
“Naparo”	MKW	Biparé	*1,010.52*	*957.2*

Tandjilé; TDJ, Moyo Kebbi East; MKE, and Mayo Kebbi West; MKW.

### Study site, experimental design and crop cultivation

Phenological and morphological traits were assessed in the fields of the experimental center of ITRAD in Youé located at 12°24′29″N Latitude North and 1°21′9,6″E Longitude East, during three consecutive years (2013; 2014 and 2015). During these years, the rainfalls were 927.5; 517.2 and 765.3 mm for 2013, 2014 and 2015 respectively. The experiments were laid out in an alpha lattice design and replicated five times. In each replication, cultivars were sown on a line in 16 seed holes, with 0.2 m between plants and 1.2 m row spacing. To minimize edge effect, three additional lines of fills were planted around each block.

The seeding plots were set up at the begining of June to the end of July. Plots were initially primed and managed for weeds. Seeds were treated with insecticide and fungicides before seeding. The seedlings were uprooted 40 to 45 days after sowing and transplanted on the same day. Block were continuously managed for weeds.

### Data collection

Two types of data, qualitative and quantitative were collected. For quantitative traits, at the late vegetative stage, the total number of leaves (TNL) was counted. At the reproductive stage, the number of days to booting (NBT), number of days to appearance of flag leaf (NFL), number of days to heading (NHD) and number of days to flowering (NFW) were recorded. At maturity, the following traits were collected: perultimate leaf length (PLL; base of leaf to leaf tip), perultimate leaf width (PLW; at widest part of leaf), perultimate leaf sheath length (PSL), internode length (INL), peduncle length (PDL; ligule of flag leaf to base of panicle), plant height (PHT, base of plant to tip of panicle), number of internodes (NIN), stem diameter (SDI; at 30 cm from plant base), number of green leaves (NGL; leaf is considered green if more than 75% of leaf is green), panicle length (PAL; base to tip of panicle), panicle width (PAW; at broadest part of panicle), weight of the main panicle (PWT; weight of main after sun and air drying for 10 days), weight of grains of the main panicle (PGW), hundred grain weight (HGW), and potential yield (PYI; as tons of grain per hectare).

For qualitative traits, the surveys were based on direct observations, with variants defined for each trait. At seedling stage, seedling color and seedling vigor were observed, 15 days after emergence. Seedling vigor rated from 1 to 9 according to stem size, leaf thickness and length of seedlings. 1 indicated poor vigor, 3; weak vigor, 5; good vigor, 7; very good vigor, and 9; excellent vigor. At maturity, the following traits were observed: panicle compactness, glume color, grain color, botanical race according to Harlan and De Wet key (1972), aristation, glume hairiness, endodermal texture estimated by BONO scale, and staygreen (STG; as percent of green leaves at maturity to total number of leaves produced).

### Statistical analysis

Variance analysis was performed to verify significance differences between cultivars for all traits. The coefficient of variation allowed to assess the levels of variation of the average observed between cultivars for each variable trait. Pearson’s R coefficient was used to measure correlations between traits. Based on the correlation of the traits, the Principal Component Analysis (PCA) allowed a small number of uncorrelated linear combinations to be selected. These were used to build the dendrogram from the Ascending Hierarchical Classification (AHC). The classes formed by AHC were used to make Discriminating Factor Analysis (DFA) which allowed groups to form by projecting both characters and individuals on a plane in order to assess the dispersion of cultivars and to better compare the variability between them. The traits used for group characterization were analyzed by ANOVA to see their involvement in the characterization and their significance.

For all traits, genetic parameters were estimated from the components of the analysis of variance. Genotypic and phenotypic variances (GV and PV), coefficients of genotypic and phenotypic variation (GCV and PCV) and heritability (H^2^) were calculated according to the formula used by^26,27^, and^28^. GenStat 12^th^ edition, XLSTAT-Pro 2016 and Excel 2010 software were used for these different analyses.

## Results

### Assessment of agro-morphological traits

Qualitative trait analysis (Table 2) showed significant genetic variation among cultivars for almost all traits except for the botanical race, which was *dura* for all cultivars. At seedling stage, 22.9% of seedlings were rated as having very good vigor while 43.8% were rated as having good vigor. Seedling color ranged from including white (35.4%), red (31.3%), green (27.1%) and yellow (6.3%). At maturity, most cultivars had compact panicles (66.7%), mostly black glumes (66.7%), and glume hairiness (58.3%), but no aristation (91.67%). Almost 62% of cultivars had white grains and 93.8% of these were vitreous.

**Table 2:** Modeling qualitative traits for dry-season sorghum collection.

Traits analysis	Modality	Frequency (%)
Color of seedling	Green	27.1
	White	35.4
	Yellow	63.0^*^
	Red	31.3
Vigor of seedling	Bad vigor	2.1
	Weak vigor	12.5
	Good Vigor	18.8
	Very good vigor	43.8^*^
	High vigor	22.9
Color of glumes	Black	66.7^*^
	Straw color	25.0
	Red	8.3
Color of grains	White	62.5^*^
	Red	25.0
	Yellow	12.5
Endodermal texture	vitreous	93.8
	Floury	6.3
Compactness of panicle	Compact	66.7^*^
	Semi-compact	33.3
Botanical race	*Durra*	100.0
Aristation	Present	8.3
	absent	91.7^*^
Hairiness of glume	Present	58.3^*^
	Absent	41.7

*Indicates significance at 0.1% probability.

Analysis of variance of the collection (Table 3) showed significant differences (P < 0.001) in all assessed quantitative traits except stem diameter (SDI). Analysis of variance by cultivars origin showed that five traits discriminated cultivars at the 0.1% probability threshold, eight traits discriminated cultivars at the 1% probability and one trait discriminated them at the 5% probability. Seven traits showed non-significant differences in cultivar origins. Coefficients of variation (CV) for most traits were below 30%, showing little variations between cultivars. However, four traits; main panicle weight (PWT), main panicle grain weight (PGW), staygreen (STG) and potential yield (PYI) showed CV above 30%, indicating a large variation among cultivars for these traits. The cultivars had between 18 to 30 leaves, with plant heights of 1.0 to 2.7 m. Number of days to flowering (NFW) ranged from 132 to 148 days. Staygreen (STG) ranged from 18 to 80%, and potential yields of 0.30 to 1.68 t/ha were observed.

**Table 3:** Analysis of variance for 22 quantitative traits for dry-season sorghum cultivars in Chad.

Characters	Min.	Max.	Average	CV (%)	Collection		Landraces Origin	
					F	R^2^ (%)	F	R^2^ (%)
TNL	18	30	23	10.0	2.7^***^	41.6	6.4^**^	3.87
PLL (cm)	53	76	64	8.5	3.0^***^	43.7	8.2^***^	5.81
PLW (cm)	5.7	10.5	8.5	11.2	3.3^***^	46.1	4.4^ns^	3.22
PSL (cm)	13	36	19	18.5	2.3^***^	37.6	4.7^**^	3.46
INL (cm)	20	35	26	13.0	3.4^***^	47.0	4.8^**^	3.51
PDL (cm)	21	52	33	21.6	10.1^***^	72.3	19.7^***^	12.96
SDI (mm)	2.9	8.2	4.4	20.0	1.1^ns^	21.6	2.6^ns^	1.94
PHT (cm)	95	272	172	22.1	11.1^***^	74.3	11.9^***^	8.25
NIN	9	23	14	17.1	7.1^***^	64.8	3.7^ns^	2.72
NGL	2.6	10.6	6.1	29.5	2.7^***^	41.4	11.5^***^	8.00
PAL (cm)	12	36	19	29.1	8.0^***^	67.6	9.9^***^	6.98
PAW (cm)	6.2	10.8	7.9	11.4	3.2^***^	45.1	2.8^ns^	2.05
NBT (days)	125	139	132	2.4	2.3^***^	37.8	7.4^**^	5.30
NFL (days)	127	142	136	2.3	1.8^**^	31.8	5.8^**^	4.19
NHD (days)	131	147	140	2.5	2.1^***^	35.4	6.9^**^	4.92
NFW (days)	132	148	142	2.6	2.2^***^	36.1	7.4^**^	5.30
PWT (g)	250	1120	624	31.8	2.4^***^	38.4	0.3^ns^	0.20
PGW (g)	150	840	437	37.3	2.7^***^	41.1	0.5^ns^	0.36
HGW (g)	2.6	6.3	4.7	18.6	—	—	—	—
STG (%)	18	81	45	31.3	2.4^***^	38.7	4.7*	3.40
PYI (t/ha)	0.30	1.68	0.87	37.3	2.7^***^	41.1	0.5^ns^	0.36

Significance: ^*^P < 0.05; ^**^P < 0.01; ^***^P < 0.001; ns: not significant; Min.: minimum; Max.: maximum; TNL: total number of leaves; PLL: perultimate leaf length; PLW: perultimate leaf width; PSL: perultimate leaf sheath length; INL: internodes length; PDL: peduncle length; SDI: stem diameter; PHT: plant height; NIN: number of internodes; NGL: number of green leaves; PAL: panicle length; PAW: panicle width; NBT: number of days to booting; NFL: number of days to flag leaf appearance; NHD: number of days to heading; NFW: Number of days to flowering; PWT: weight of the main panicle; PGW: grain weight of main panicle; HGW: hundred grain weight; STG: staygreen; PYI: potential yield.

Analysis of average performance of cultivars by region (Table 4) provided an assessment of the variability among cultivars in their region. For the Mayo Kebbi Est (MKE) region, the CV were at or less than 30% for most traits except PAL (32%), reflecting low variation among cultivars in this region. For the Mayo Kebbi West (MKW) region, the CV were less than 30% except for PGW and PYI, reflecting a small variation among cultivars in this region. Finally, for the Tandjilé region, PWT, PGW, STG, and PYI had CV above 30%, indicating a significant variation amongst cultivars in this region.

**Table 4:** Average performances of dry-season sorghum cultivars for each geographical region.

Traits	Tandjilé (22 accessions)				Mayo Kebbi East^35^				Mayo Kebbi West^38^			
	Min.	Max.	Ave.	CV	Min.	Max.	Ave.	CV	Min.	Max	Ave	CV
TNL	18	28	23	9.6^*^	18	25	22	9.3^***^	21	30	23	9.8^***^
PLL	53	70	62	8.5^**^	58	76	67	8.0^***^	56	73	63	7.3^***^
PLW	5.7	10.5	8.0	14.2^***^	7.2	9.8	8.5	9.0^*^	7.1	9.9	8.8	8.3^*^
PSL	13	23	19	12.3^***^	14	36	21	23.7^ns^	16	24	19	14.5^***^
INL	20	33	25	11.6^**^	20	3	28	12.4^***^	22	35	27	14.2^***^
PDL	24	44	31	18.2^***^	27	52	37	21.6^***^	21	40	30	18.1^***^
SDI	3.3	8.0	4.7	24.7^ns^	3.0	6.0	4.0	16.0^ns^	3.0	5.0	4.0	13.6 ^ns^
PHT	95	228	156	18.5^***^	100	225	183	20.0^***^	123	272	180	24.7^***^
NIN	11	17	14	12.5^***^	10	18	14	16.7^***^	9	23	15	21.7^***^
NGL	2.7	8.3	5.5	28.6^*^	3.0	10.7	5.9	29.9^***^	3.5	10.1	7.3	23.1^*^
PAL	13	27	19	23.6^***^	14	36	22	31.8^***^	12	31	18	26.3^***^
PAW	6.3	10.5	8.2	12.6^***^	6.9	10.9	7.9	12.2^***^	6.2	8.5	7.7	7.5^ns^
NBT	127	139	132	2.5^***^	125	136	131	2.2^**^	129	138	134	2.1 ^ns^
NFL	132	141	136	2.0 ^ns^	127	140	134	2.4^**^	132	142	137	1.9 ^ns^
NHD	134	144	139	2.0 ^ns^	132	144	138	2.7^***^	135	147	142	2.4^***^
NFW	135	147	141	2.2 ^ns^	132	145	140	2.7^**^	137	148	144	2.4^**^
PWT	340	1120	621	39.7^***^	410	960	640	26.6^*^	250	880	610	27.3 ^ns^
PGW	160	840	425	48.9^***^	262	740	451	29.6^*^	150	680	439	30.4 ^ns^
HGW	3.0	6.2	4.6	18.1	3.5	6.3	5.0	17.5	2.6	6.3	4.6	20.5
STG	18	66	41	32.9^*^	18	66	44	28.9^***^	21	81	52	28.6^**^
PYI	0.3	1.7	0.9	48.9^***^	0.5	1.5	0.9	29.6^*^	0.3	1.4	0.9	30.4 ^ns^

Significance: ^*^P < 0.05; ^**^P < 0.01; ^***^P < 0.001; ns: not significant; Min: minimum; Max: maximum; TNL: total number of leaves; PLL (cm): perultimate length leaf; PLW (cm): perultimate leaf width; PSL (cm): perultimate leaf sheath length; INL (cm): internodes length; PDL (cm): peduncle length; SDI (mm): stem diameter; PHT (cm): plant height; NIN: number of internodes; NGL: number of leaves remaining green; PAL (cm): panicle length; PAW (cm): panicle width; NBT (days): number of days to booting; NFL (days): number of days to flag leaf appearance; NHD (days): number of days at heading; NFW (days): Number of days at flowering; PWT (g): weight of the main panicle; PGW (g): weight of main panicle grains; HGW (g): 100 grain weight; STG (%): staygreen; PYI (t/ha): potential yield.

Analysis of minimum and maximum values for traits by region showed that the the days to flowering ranged from 135–148 days across three regions. Potential yield ranged from 0.3–1.7 ton/ha, with the maximum yield recorded at Tandjilé. On average, cultivars produced heavier main panicles (640 g) and main panicle grain yield (451 g) in Mayo Kebbi East. Average staygreen was highest (52%) in Mayo Kebbi west, which also had the highest average number of green leaves (7.3) at maturity.

Analysis of variance across region showed significative differents for most assessed quantitatives traits (P < 0.001). In the Tandjile region, only four traits showed a non-significant difference between accessions; in the Mayo Kebbi East, only two traits shown a non significative difference between accessions and in the Mayo Kebbi West seven traits shown non significant difference between accessions of this group. Only stem diameter and hundred grain weight showed non-significant difference amongst cultivars in all three regions. Meanwhile, total number of leaves, perultimate leaf length, perultimate leaf width, internode length, peduncle length, plant height, number of internodes, number of green leaves at maturity, panicle length, and the staygreen traits were significantly different amongst cultivars in all zones.

### Correlation analysis among traits

The Pearson test (Table 5) showed that potential yield was positively influenced by length of the perultimate leaf (r = 0.26), perultimate leaf width (r = 0.75), number of green leaves (r = 0.30) and the weight of the main panicle (r = 0.90). However, longer peduncle length (r = −0.34), perultimate leaf sheat length (r = −0.24), and separation (r = −0.23) negatively impacted potential yield. Cultivars with more internodes flowered earlier (r = 0.36), while those with longer peduncles flowered late (r = −0.38). Taller cultivars tend to have longer (r = 0.82) and more (r = 0.66) internodes. Cultivars that showed the staygreen trait had fewer internodes (r = −0.41), were generally shorter (r = −0.25) with broader perultimate leaf width (r = 0.38) and more green leaves at maturity (r = 0.82).

**Table 5:** Pearsons phenotypic correlation coefficient of 17 quantitative traits studies for dry-season sorghum in Chad.

Traits	PLL	PLW	PSL	INL	PDL	SDI	PHT	NIN	NGL	PAL	PAW	NHD	NFW	PWT	PGW	STG
PLW	0.20^*^															
PSL	0.43^**^	−0.26^*^														
INL	0.36^**^	−0.14	0.63^**^													
PDL	0.48^**^	−0.27*	0.64^**^	0.48^**^												
SDI	−0.07	−0.22^*^	−0.01	−0.12	0.15											
PHT	0.43^**^	−0.11	0.52^**^	0.82^**^	0.39^**^	−0.07										
NIN	0.17	0.05	0.03	0.36^**^	−0.27^*^	−0.07	0.66^**^									
NGL	0.00	0.45^**^	−0.10	0.09	−0.19	−0.13	0.15	0.16								
PAL	0.58^**^	0.05	0.55^**^	0.49^**^	0.76^**^	0.10	0.52^**^	0.00	−0.04							
PAW	0.23*	0.56^**^	0.05	0.02	−0.12	−0.05	0.14	0.25^*^	0.17	0.36						
NHD	−0.12	0.05	0.02	0.11	−0.33^**^	−0.06	0.20^*^	0.36^**^	0.13	−0.14	0.07					
NFW	−0.15	0.05	0.00	0.07	−0.38^**^	−0.10	0.15	0.36^**^	0.09	−0.20^*^	0.06	0.97^**^				
PWT	0.27*	0.72^**^	−0.19	−0.12	−0.30^**^	0.03	0.01	0.27^*^	0.28^**^	0.04	0.55^**^	−0.06	−0.09			
PGW	0.26*	0.75^**^	−0.23^*^	−0.14	−0.34^**^	−0.01	−0.03	0.25^*^	0.30^**^	−0.02	0.54^**^	−0.01	−0.04	0.98^**^		
STG	−0.11	0.38^**^	−0.11	−0.14	−0.05	−0.09	−0.25^*^	−0.41^**^	0.82^**^	−0.09	−0.01	−0.05	−0.09	0.10	0.13	
PYI	0.26^*^	0.75^**^	−0.23*	−0.14	−0.34^**^	−0.01	−0.03	0.25^*^	0.30^**^	−0.02	0.54^**^	−0.01	−0.04	0.98^**^	1.00	0.13

Significance at ^*^P < 0.05; ^**^P < 0.01; Min.: minimum; Max.: maximum; PLW: perultimate leaf width; PSL: perultimate leaf sheath length; INL: internodes length; PDL: peduncle length; SDI: stem diameter; PHT: plant height; NIN: number of internodes; NGL: number of green leaves at maturity; PAL: panicle length; PAW: panicle width; NHD: number of days to heading; NFW: Number of days to flowering; PWT: weight of the main panicle; PGW: weight of the grains of the main panicle; STG: staygreen; PYI: potential yield.

### Principal component analysis of various traits

The Principal Components Analysis (PCA) with all studied traits showed that the first 5 components accounted for 80.1% of the total variability (Table 6). Axis 1 with 23.35% of total variability was defined by the number of days to flowering, number of days to heading, number of days to booting, the number of days to appearance of the flag leaf, and the peduncle length. This axis was related to the cultivars’ developmental cycle. Axis 2 with 22.74% of variability total was associated with days to flowering, potential yield, perultimate leaf width, hundred grain weight, main panicle weight, number of days to booting, and number of days to flag leaf appearance. This axis reflected cultivar cycle (w.r.t. number of days to booting, flag leaf appearance, and flowering) and performance (w.r.t. panicle weight, hundred grain weight and potential yield). Axis 3 with 19.56% of total variability was defined by plant height, perultimate leaf length, internode length, number of internodes, panicle length, and panicle width. This axis was related to plant architecture. Axis 4 with 8.42% of total variability was strongly correlated with the staygreen and the number of green leaves at maturity. This axis defined cultivars’ drought tolerance. Finally, the axis 5 with 5.98% of total variability was defined by the stem diameter and panicle length.

**Table 6:** Principal component analysis (PCA) of different quantitative agro-morphological traits.

Principal Component	F1	F2	F3	F4	F5
Eigenvalue	5.12	5.00	4.30	1.85	1.32
% total variance	23.35	22.74	19.56	8.42	5.98
Cumulative variance %	23.35	46.09	65.65	74.07	80.05
PLL	3.33	0.66	7.81	0.34	2.64
SDI	0.18	4E-04	0.52	3.99	11.79
PHT	2.01	2.806	14.14	0.01	5.04
NIN	1.32	1.04	9.79	5.98	14.75
PAL	7.38	0.01	6.38	0.19	13.11
PAW	0.65	2.85	7.22	0.69	6.33
NFW	8.39	6.99	1.84	0.32	4.37
HGW	1.13	7.07	0.45	1.68	0.08
STG	0.47	2.356	0.23	42.42	0.03
PYI	3.02	10.39	5.31	1.75	0.11
PWT	3.018	10.39	5.31	1.75	0.11
PLW	2.68	9.23	3.54	2.29	1.41
PSL	6.83	1.96	4.27	1.00	2.94
INL	4.08	2.84	9.44	1.12	3.65
PDL	14.57	0.28	0.58	1.17	7.49
NLG	1.64	1.34	2.12	31.15	4.08
NBT	7.45	8.78	0.08	0.61	5.02
NFL	8.00	7.68	1.40	0.30	5.14
NHD	7.96	6.88	2.57	0.61	5.02
PGW	2.23	10.41	5.68	2.34	0.05

PLL: perultimate length leaf; SDI: stem diameter; PHT: plant height; NIN: number of internodes; PAL: panicle length; PAW: panicle width; NFW: Number of days to flowering; HGW: 100 grain weight; STG: staygreen; PYI: potential yield; PWT: weight of the main panicle; PLW: perultimate leaf width; PSL: perultimate leaf sheath length; INL: internodes length; PDL: peduncle length; NGL: number of green leaves at maturity; NBT: number of days to booting; NFL: number of days to appearance of flag leaf; NHD: number of days to heading; PGW: weight of the grains of the main panicle.

### Morphological cluster analysis

**Figure 1:**
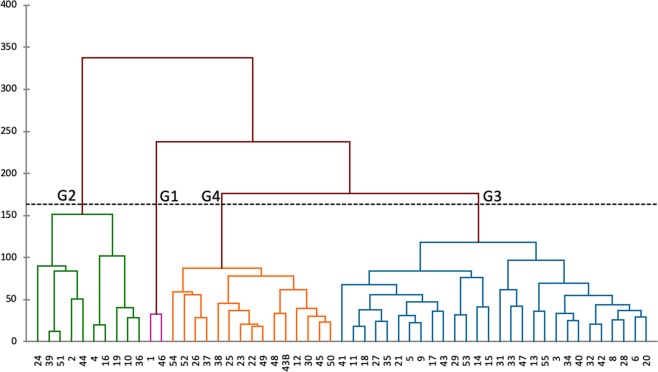
Dendrogram showing clustering pattern of 54 genotypes of Chadian dry-season sorghum on the basis of genetic their divergence. (G1: group 1; G2: group 2; G3: group 3; G4: group 4).

Hierarchized Ascending Classification (HAC) was performed using Euclidean distances with the average link method as an aggregation criterion and an intergroup variance of 81.54% of the total variance (Fig. 1). Ten least correlated variables were used for this purpose, and automatic truncation at the inertial 170 level allowed cultivars to be clustered into four very distinct groups in the dendrogram: the first group had two cultivars, the second group had 10 cultivars, the third group had 28 cultivars and fourth group had 15 cultivars.

Analysis of Fisher distances between groups (Table 7) showed the distances were higher between group 2 and group 3 (26.2), followed by the group 1 and group 2 (12.4), then group 3 and group 4 (8.9), group 2 and group 4 (7.4), and group 1 and group 4 (5.4). There were significant differences (P < 0.001) between all the groups, except between group 1 and group 3.

**Table 7:** Fisher distances and significant difference between groups.

Group	1	2	3
2	12.4^***^		
3	2.0^ns^	26.2^***^	
4	5.4^***^	7.4^***^	8.9^***^

^***^Indicates significance at 0.1% probability; ns: difference non significant.

**Figure 2:**
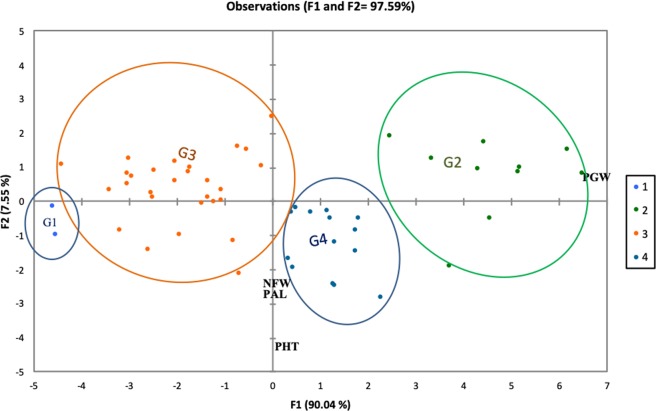
Discriminant factor analysis showing four major groupings of Chadian dry-season sorghum cultivars based on inter-trait commonality.

Discriminant function analysis (DFA) showed the position of individual groups on the 1/2 axis with a total inertia of 97.59% (Fig. 2). Function 1 with inertia of 90.04% was defined by plant height, panicle length and number of days to flowering. Function 2 with 7.55% inertia was defined by grain weight of the main panicle. The Wilks Lambda test (Rao approximation) gave observed F of 6.14 while the critical F was 1.55. The groups were therefore different entities from each other, except between the group 1 and group 3, where the difference was non significant (Table 7). The position of the individual cultivar on the DFA 1/2 axes made it possible to characterize the groups. Group 1 was composed of the earliest cultivars, with very short panicles length and panicles width, very low panicle grain yield and the lowest staygreen. Group 2 consisted of 10 cultivars with short height, medium cycle, best main panicle grain weight, and best staygreen. This group showed very good trait combinability for selection. Group 3 consisted of medium height cultivars with a fairly long number of days to flowering and low main panicle weight. Group 4 consisted of very tall cultivars with a late flowering, long panicle length, medium panicle width, and a medium main panicle grain yield.

The analysis of the average performance of the cultivars by analysis of the variance showed that for seven traits, there were non-significant differences between the groups. However, for the main panicle weight and panicle width traits, there were high significant differences at the 0.1% probability between groups while for the number of internodes there was a significant difference at the 5% probability level between groups. These were the three characters that discriminated groups.

### Estimate of genetic parameters and performance of cultivars

Analysis of genetic parameters (Table 8) showed that genotypic variance (GV) were lower than phenotypic variance (PV) for all traits assessed exept potential yield. Indeed, the GV and PV were quite low for several traits except for main panicle weight, plant height, perultimate leaf length, main panicle weight and staygreen rating. A relative amount of variation in genotypes for traits can be judged by comparing the coefficient of genotypic (GCV) and phenotypic (PCV) variations. The phenotypic coefficient of variation was higher than genotypic coefficient of variation for all traits. Higher magnitudes of GCV and PCV occurred for potential yield (29.6% and 37.1%), followed by grain weight of the main panicle (29.3% and 36.8%), staygreen (26.0% and 33.2%), main panicle weight variables (24.2% and 31.4%), number of green leaves at maturity (23.2% and 29.2%), panicle length (27.0% and 28.9%), plant height (20.6% and 21.6) and peduncle length (20.1% and 21.2%) respectively.

**Table 8:** Calculated genetic parameters of dry-season sorghum cultivars.

Traits	GV	PV	H^2^	√VG	√VP	GCV (%)	PCV (%)
NBT	6.5	11.2	58.4	2.6	3.4	1.9	2.5
PHT	1256	1379	91.1	35.5	37.1	20.6	21.6
PLW	0.6	0.9	69.0	0.8	0.9	9.2	11.1
PAW	0.6	0.8	68.1	0.74	0.9	9.3	11.3
INL	7.9	11.2	70.2	2.8	3.3	10.6	12.6
PLL	19.0	29.6	64.2	4.4	5.4	6.8	8.5
PSL	7.3	12.6	57.8	2.7	3.6	13.9	18.3
PAL	27.8	31.8	87.5	5.3	5.6	27.0	28.9
PDL	43.2	47.9	90.2	6.6	6.9	20.1	21.2
NIN	4.7	5.4	86.3	2.2	2.3	15.6	16.8
NGL	2.0	3.2	63.4	1.4	1.8	23.2	29.2
NHD	6.9	12.6	54.6	2.6	3.5	1.9	2.5
NFW	7.5	13.5	55.7	2.7	3.7	1.9	2.6
PGW	16442.0	25988.0	63.3	128.2	161.2	29.3	36.8
PWT	22826.0	38484.0	59.3	151.1	196.2	24.2	31.4
PYI	0.1	0.1	63.6	0.3	0.3	29.6	37.1
NFL	4.4	9.5	45.9	2.1	3.1	1.5	2.3
STG	137.3	223.3	61.5	11.7	14.9	26.0	33.2

NBT: number of days to booting; PHT: plant height; PLW: perultimate leaf width; PAW: panicle width; INL: internodes length; PLL: perultimate length leaf; PSL: perultimate leaf sheath length; PAL: panicle length; PDL: peduncle length; NIN: number of internodes; NGL: number of green leaves at maturity; NHD: number of days to heading; NFW: Number of days to flowering; PGW: weight of the grains of the main panicle; PWT: weight of the main panicle; PYI: potential yield; NFL: number of days to appearance of flag leaf; STG: staygreen.

Selection is favored when a major proportion of a large amount of phenotypic variability is due to heritable variation. In this study, heritability ranged from 45.9 to 91.1%. In fact, heritability was highest (H^2^ > 82) for plant height (91.1), panicle length (87.5), peduncle length (90.2), number of internodes (86.3), and separation (82.9). High heritability values (51% < H^2^ < 70%) were observed for staygreen (61.2), potential yield (63.6), and all other traits except for the number of days to appearance of flag leaf which showed average heritability (45.9).

Analysis of average cultivar yield performance (Table 9) showed ten of the most productive landraces in the collection, with potential yields ranging from 1.2 to 1.7t/ha. The landrace “Farine”, native to Tandjilé showed the best production potential with the highest potential yield (1.68t/ha), medium plant height (1.4 m), a comparatively shorter number of days to flowering (138days), and a very high stay green potential (62%). It was followed by the “Gogmi Rouge” with similar yield potential (1.66t/ha), good staygreen (55%) but taller plants (1.6 m). “Gogoumi” of similar height to Farine, yielded 1.52 t/ha with 51% staygreen. “Glinding” and “Gogoum jaune” which were the shortest (1.2 m) cultivars, yielded 1.48 and 1.43t/ha with 43.4 and 47.4% staygreen respectively. “Vounging Membou” which had the highest staygreen (63%), shortest days to flowering (137days) had a comparatively lower potential yield (1.2t/ha).

**Table 9:** Combinabilty performance of dry-season sorghum cultivars in Chad.

Cultivars	PHT (cm)	NFW (days)	STG (%)	PYI (t/ha)	Origin
“Farine”	142.8	138.6	61.81	1.68	Tandjilé
“Gogmi rouge”	157.8	138.2	55.1	1.7	Tandjilé
“Gogoumi”	137.8	142.2	50.5	1.5	Tandjilé
“Glinding”	116.5	139.4	43.4	1.5	Mayo Kebbi Est
“Gogoum jaune”	122.0	139.5	47.4	1.4	Tandjilé
“Donglon rouge”	190.3	143.4	41.8	1.4	Mayo Kebbi Est
“Dalassi”	184.5	147.4	40.9	1.4	Mayo Kebbi Ouest
“Farine Kouamgue”	157.5	141.0	31.2	1.3	Tandjilé
“Tchokowawana”	225.0	140.0	29.9	1.2	Mayo Kebbi Est
“Vounging Membou”	133.3	137.0	62.9	1.2	Mayo Kebbi Ouest

PHT: plant height; NFW: number of days to flowering; STG: staygreen; PYI: potential yield.

## Discussion

The study showed that dry-season sorghum landraces from the Sudanese zone of Chad were all from *durra* race. Dry-season sorghums are considered to be *durra*^29^; or they are classified as durra or *caudatum* or *dura-caudatum* hybrids^30^; or *durra* and *guinea* race^31^. It was also found that these cultivars have white seeds (62.5%) of which 93.7% were vitreous. According to^32^ farmers’ demand for new varieties is higher for white grains sorghum, which has a wider appeal for preparation of traditional meals. As vitrosity is a highly appreciated trait by farmers, the high vitrosity of these cultivars could be used to enhance this trait. The study showed that most cultivars had compact panicles (66.6%), mostly black glumes (66.6%) and glume hairiness (58.3%) and did not have aristation (91.6%). According to^22^ dry-season sorghum have very large stems, reduced tillering, compact panicles, often crossed peduncle, probably selected by farmers because it facilitates protection against bird attacks and their grain is big with vitreous albumen.

The coefficients of variation for most traits were below 30%, which indicated small amplitude of variation in the traits relative to the mean value. This would show a relatively low genetic diversity of dry-season sorghum. Indeed, according to^10^, oral traditions reported a process of “seasonal adjustment” about the origin of dry-season sorghums, which consisted of selecting varieties adapted to the dry season from rain-season sorghums. From genetic point of view, this approach may have caused a bottleneck, leading to a decreased level of diversity in dry-season germplasm, especially if only a limited portion of the rainfed sorghum population was used in this process^30^. Selected for centuries to be cultivated in very harsh climatic environments, transplanted sorghum has traits that differentiate them from sorghum grown during the rainy season^22^. However, even though dry-season sorghum was derived from the selection of rainfed sorghum and has been cultivated for several centuries, there still exist some genetic diversity among cutivars. In fact, the analysis of variance showed that all traits discriminated cultivars at the 0.1% probability threshold except for one trait, which reflected non significant genetic variability among cultivars.

Analysis of the average performance of cultivars by region shows high variability within and between regions. Cultivars from Mayo Kebbi West had a long average number of days to flowering and cultivars from Tandjilé have the highest average potential yield. The evaluation of the average of traits gave a staygreen with a minimum of 18% and a maximum of 81% at maturity. This means that during grain filling, cultivars kept at least 18% to 81% of their total number of leaves green. It should be noted that all cultivars were from the *durra* race and according to^17^, the most common source of staygreen has historically been the sorghum line, BTx642 (formerly called B35), a member in the *durra* race.

The study showed that staygreen positively influenced potential yield. Staygreen was high for short cultivars with low numbers of internodes and broader perultimate leaves, which was a significant contributor to potential yield. Principal component analysis showed a link between number of days to flowering, staygreen, perultimate leaf width and yield potential.

Through ten less correlated traits, the structuring of diversity allowed cultivars to be clustered into four statistically distinct groups except for groups 2 and 3, which were non significantly different. Group 2 cultivars were shorter with medium number of days to flowerings, better staygreen and better seed production of the main panicle. The combinability of high productivity, precocity, and the staygreen, all which contribute to cultivar performance, suggest cultivars of this group could be of highest interest to breeding programs. The estimation of genetic parameters showed that genotypic and phenotypic variances were low for most of the traits. The traits with higher magnitudes of coefficient of variation offer a better opportunity for improvement through selection^33^.

However, most of the traits had fairly high heritability. Heritability broadly represents the proportion of total variability observed due to genetic variability^34^. According to^35^, heritability is high above 50%, low in declines of 20% and average between 20 and 50%. Thus, only the number of days to flag leaf appearance had a mean heritability. Plant height had highest heritability, followed by the peduncle length, the panicle length and the number of internodes.

Performance analysis of average cultivar values showed that the “Farine” cultivar, with a very short height, had the highest potential yield (1.68 t/ha), with a shorter number of days to flowering and a high stay green potential. It was followed by “Gogmi Rouge” (1.66 t/ha), then “Gogoumi” (1.52 t/ha), “Glinding” (1.48 t/ha) and “Gogoum jaune” (1.43 t/ha). According to^36^, under Mauritanian conditions yields of dry-season sorghum are low (0.35–0.40 t/ha) and very variable, and can only reach more than 1.0t/ha under semi-controlled conditions. It was observed that the potential yield of cultivars is strongly linked to their staygreen and the most productive cultivars had the largest staygreen. According to^37^, the selection for staygreen is expected to have a significant implication in productivity of wheat particularly under harsh environments.

## Conclusion

Phenotypic evaluation of germplasm can be useful for characterization, conservation and maintenance of genetic resources. This study revealed a large agro-morphological diversity of qualitative and quantitative traits. The significant and positive correlation between the staygreen and the yield potential could be used for genetic improvement of dry-season sorghum in Chad. Structuring of diversity clustered the cultivars into four groups, of which in group two were found short cultivars, with medium cycle, better seed production of the main panicle and better staygreen. This group was of interest for breeding because their cultivars combined the productivity of the panicle, shorter number of days to flowering and staygreen; traits that contribute to the performance of cultivars. The staygreen trait, which is a characteristic of resistance to dryness, seems strongly related to dry-season sorghum of Chad. The best lines with desired quantitative and qualitative traits were “Farine”, “Gogmi Rouge”, “Gogoumi”, “Glinding”, and “Gogoum jaune”. These genotypes were selected for further breeding and introgression into elite germplasm.
